# Noise During Cognitive Tests and Older Adults’ Cognitive Performance: Evidence From India

**DOI:** 10.1093/geroni/igaf122.539

**Published:** 2025-12-31

**Authors:** Yezhen Li, Clarice Myers, Wuyang Zhang, Jinkook Lee, Nicholas Reed, Emma Nichols

**Affiliations:** University of Southern California, Los Angeles, California, United States; Johns Hopkins School of Medicine Department of Otolaryngology-Head and Neck Surgery, Baltimore, Maryland, United States; Johns Hopkins Bloomberg School of Public Health, Baltimore, Maryland, United States; University of Southern California, Los Angeles, California, United States; New York University Grossman School of Medicine, New York, New York, United States; University of Southern California, Los Angeles, California, United States

## Abstract

Existing evidence suggests that chronic noise exposure elevates the risk of cognitive impairment among older adults. However, noise during cognitive testing may be associated with chronic noise exposure and could impact cognitive testing, leading to bias in analyses of the chronic noise-cognition association. The risk of such biases may be larger in developing regions, where creating optimal test conditions may be more challenging. Using data from the second wave of the Longitudinal Aging Study in India - Diagnostic Assessment of Dementia (n = 3,507), we examined the associations between interviewer-rated noise during assessment and cognitive functioning. We assessed whether the associations were explained by potential mediators linked to chronic noise exposure and cognition (e.g., depression, sleep quality) and whether they were moderated by hearing abilities. High noise levels during testing were significantly associated with lower general cognitive performance scores (b = -.309; p < .001) and lower scores in several cognitive domains, including executive functioning, language, visuospatial, and memory (b = -.280, -.398, -.156, and -.254; p < .05). Associations remained after accounting for potential mediators of chronic noise-cognition associations, suggesting observed effects may be due to acute impacts of noise during assessment rather than chronic noise exposures. Moreover, the moderating effects of hearing abilities were curvilinear, with stronger associations for respondents with normal hearing range and severe hearing loss. These findings suggest that testing conditions, including noise, may factor into older adults’ cognitive assessment results. Further research is needed to better understand the implications of noise during cognitive tests.

